# Supplementary material for: Entomological surveys and insecticide resistance in the dengue vector Aedes aegypti in Dakar, Senegal: First detection of the kdr mutation
Source: PLoS Negl Trop Dis. 2025 Oct 22;19(10):e0013657. doi: 10.1371/journal.pntd.0013657 (PMC12561948; doi:10.1371/journal.pntd.0013657)
Supplement: S1 Table — (DOCX) [file pntd.0013657.s001.docx]

**S1 Table.** Number of adult mosquitoes collected from August 2022 to July 2023 in Dakar

| **Localities** | **Aspiration** | **BG Lure + CO2** |  | **Total** |
| --- | --- | --- | --- | --- |
| **Species** |  | **Day** | **Night** |  |
| **Grand Yoff** | **1539** | **82** | **204** | **1825** |
| *Aedes aegypti* | 111 | 20 | 6 | 137 |
| *Anopheles gambiae* s.l. | 2 | 0 | 0 | 2 |
| *Anopheles pharoensis* | 0 | 0 | 1 | 1 |
| *Culex quinquefasciatus* | 1423 | 62 | 196 | 1681 |
| *Anopheles sp* | 0 | 0 | 1 | 1 |
| *Culex theileri* | 1 | 0 | 0 | 1 |
| *Culex tritaeniorhynchus* | 2 | 0 | 0 | 2 |
| **Guédiawaye** | **1007** | **1521** | **9874** | **12402** |
| *Aedes aegypti* | 66 | 42 | 22 | 130 |
| *Culex cinereus* | 0 | 0 | 2 | 2 |
| *Anopheles gambiae* s.l. | 14 | 39 | 72 | 125 |
| *Culex quinquefasciatus* | 926 | 1437 | 9772 | 12135 |
| *Culex tritaeniorhynchus* | 1 | 3 | 6 | 10 |
| **Mbao** | **1927** | **1360** | **10003** | **13290** |
| *Aedes aegypti* | 113 | 24 | 25 | 162 |
| *Mansonia africana* | 3 | 153 | 881 | 1037 |
| *Culex antennatus* | 0 | 0 | 1 | 1 |
| *Culex cinereus* | 0 | 2 | 5 | 7 |
| *Anopheles gambiae* s.l. | 6 | 7 | 62 | 75 |
| *Culex neavei* | 1 | 0 | 0 | 1 |
| *Culex nebulosus* | 0 | 0 | 4 | 4 |
| *Culex perfuscus* | 1 | 0 | 3 | 4 |
| *Anopheles pharoensis* | 0 | 0 | 1 | 1 |
| *Culex poicilipes* | 0 | 1 | 5 | 6 |
| *Culex quinquefasciatus* | 1762 | 996 | 8099 | 10857 |
| *Anopheles rufipes* | 0 | 0 | 2 | 2 |
| *Anopheless* sp | 0 | 1 | 3 | 4 |
| *Culex theileri* | 0 | 0 | 8 | 8 |
| *Culex tritaeniorhynchus* | 24 | 125 | 542 | 691 |
| *Mansonia uniformis* | 17 | 51 | 362 | 430 |
| **Médina** | **476** | **230** | **912** | **1618** |
| *Aedes aegypti* | 185 | 92 | 44 | 321 |
| *Anopheles gambiae* s.l. | 3 | 0 | 8 | 11 |
| *Culex quinquefasciatus* | 282 | 138 | 857 | 1277 |
| *Culex tritaeniorhynchus* | 1 | 0 | 3 | 4 |
| *Mansonia uniformis* | 5 | 0 | 0 | 5 |
| **Ouakam** | **89** | **217** | **2185** | **2491** |
| *Aedes aegypti* | 22 | 54 | 35 | 111 |
| *Anopheles gambiae* s.l. | 0 | 0 | 1 | 1 |
| *Culex quinquefasciatus* | 67 | 163 | 2149 | 2379 |
| **Point E** | **888** | **807** | **1345** | **3040** |
| *Aedes aegypti* | 352 | 456 | 290 | 1098 |
| *Culex cinereus* | 0 | 1 | 0 | 1 |
| *Anopheles gambiae* s.l. | 22 | 3 | 59 | 84 |
| *Culex quinquefasciatus* | 511 | 346 | 993 | 1850 |
| *Anopheles* sp | 0 | 1 | 1 | 2 |
| *Culex tritaeniorhynchus* | 3 | 0 | 2 | 5 |
| **Total** | **5926** | **4217** | **24523** | **34666** |
