# Supplementary material for: Entomological surveys and insecticide resistance in the dengue vector Aedes aegypti in Dakar, Senegal: First detection of the kdr mutation
Source: PLoS Negl Trop Dis. 2025 Oct 22;19(10):e0013657. doi: 10.1371/journal.pntd.0013657 (PMC12561948; doi:10.1371/journal.pntd.0013657)
Supplement: S2 Table — (DOCX) [file pntd.0013657.s002.docx]

S2 Table. Mean number of mosquitoes species collected per localities per month in Dakar from August 2022-July2023

| Localities | Mean | 95% CI |
| --- | --- | --- |
| Grand Yoff | 2.39 | (1.33-4.32)b |
| Guédiawaye | 2.50 | (1.40-4.46)b |
| Mbao | 6.31 | (4.26-9.34)a |
| Médina | 2.50 | (1.40-4.46)b |
| Ouakam | 2.07 | (1.10-3.88)b |
| Point E | 2.94 | (1.71-5.03)b |

(a,b) indicate that the mean number of mosquitoes species for these localities are significantly different,
