# Supplementary material for: Entomological surveys and insecticide resistance in the dengue vector Aedes aegypti in Dakar, Senegal: First detection of the kdr mutation
Source: PLoS Negl Trop Dis. 2025 Oct 22;19(10):e0013657. doi: 10.1371/journal.pntd.0013657 (PMC12561948; doi:10.1371/journal.pntd.0013657)
Supplement: S3 Table — (DOCX) [file pntd.0013657.s003.docx]

**S3 Table.** Knockdown times (KDT₅₀ and KDT₉₅, with 95% confidence intervals) of *Aedes aegypti* following exposure to different concentrations of pyrethroids across six localities in Dakar/ August 2022 to July 2023

| **Site** | **Insecticide** | **KDT_50_** | **KDT_95_** | **KDT_50__IC95** | **KDT_95__IC95** |
| --- | --- | --- | --- | --- | --- |
| Ouakam | Alphacypermethrin (0.08%) | 36 | 150 | 8.45-414 | 26-2711 |
| Ouakam | Alphacypermethrin (0.25%) | 30 | 66 | 13-88 | 27-214 |
| Ouakam | Deltamethrin (0.03%) | 80 | 163 | 16-1043 | 29-2653 |
| Ouakam | Deltamethrin (0.25%) | 25 | 70 | 6.68-207 | 16-823 |
| Ouakam | Lambdacyhalothrin (0.08%) | 53 | 102 | 19-221 | 33-477 |
| Ouakam | Lambdacyhalothrin (0.25%) | 16 | 34 | 7.07-47 | 14-119 |
| Ouakam | Permethrin (0.4%) | 103 | 252 | 8.96-25305 | 17-109209 |
| Ouakam | Permethrin (3.75%) | 10 | 18 | 3.45-73 | 5.3-160 |
| Mbao | Alphacypermethrin (0.08%) | 43 | 116 | 17-144 | 41-448 |
| Mbao | Alphacypermethrin (0.25%) | 1,49 | 25 | 0.5136-17 | 3.96-1875 |
| Mbao | Deltamethrin (0.03%) | 66 | 150 | 20-364 | 40-976 |
| Mbao | Deltamethrin (0.25%) | 6,52 | 20 | 2.55-32 | 6.33-146 |
| Mbao | Lambdacyhalothrin (0.08%) | 17 | 55 | 5.95-76 | 16-341 |
| Mbao | Lambdacyhalothrin (0.25%) | 9,91 | 30 | 4.5-31 | 12-117 |
| Mbao | Permethrin (0.4%) | 73 | 128 | 4.5-2745 | 16-6137 |
| Mbao | Permethrin (3.75%) | 5,56 | 17 | 1.82-56 | 4.15-304 |
| Medina | Alphacypermethrin (0.08%) | 27 | 91 | 6.79-285 | 18-1416 |
| Medina | Alphacypermethrin (0.25%) | 14 | 39 | 4.83-76 | 11-282 |
| Medina | Deltamethrin (0.03%) | 51 | 107 | 13-422 | 24-1069 |
| Medina | Deltamethrin (0.25%) | 15 | 36 | 4.76-108 | 9.52-344 |
| Medina | Lambdacyhalothrin (0.08%) | 60 | 125 | 18-354 | 33-865 |
| Medina | Lambdacyhalothrin (0.25%) | 28 | 68 | 13-73 | 30-199 |
| Medina | Permethrin (0.4%) | 65 | 140 | 19-375 | 36-950 |
| Medina | Permethrin (3.75%) | 8,89 | 32 | 3.08-53 | 8.51-292 |
| Guédiawaye | Alphacypermethrin (0.08%) | 35 | 94 | 15-110 | 35-339 |
| Guédiawaye | Alphacypermethrin (0.25%) | 20 | 38 | 9.29-54 | 17-116 |
| Guédiawaye | Deltamethrin (0.03%) | 273 | 1483 | 13-874608 | 43-19603647 |
| Guédiawaye | Deltamethrin (0.25%) | 19 | 47 | 8.81-49 | 20-144 |
| Guédiawaye | Lambdacyhalothrin (0.08%) | 53 | 106 | 15-337 | 27-792 |
| Guédiawaye | Lambdacyhalothrin (0.25%) | 17 | 41 | 6.67-70 | 14-202 |
| Guédiawaye | Permethrin (0.4%) | 91 | 191 | 11-6793 | 18-20457 |
| Guédiawaye | Permethrin (3.75%) | 6,4 | 14 | 1.54-464 | 2.61-2289 |
| Point E | Alphacypermethrin (0.08%) | 34 | 96 | 7.76-400 | 18-1617 |
| Point E | Alphacypermethrin (0.25%) | 28 | 55 | 8.95-155 | 16-356 |
| Point E | Deltamethrin (0.03%) | 141 | 299 | 6.22-2483173 | 10-11630836 |
| Point E | Deltamethrin (0.25%) | 26 | 72 | 7.97-155 | 19-561 |
| Point E | Lambdacyhalothrin (0.08%) | 78 | 128 | 17-769 | 26-1447 |
| Point E | Lambdacyhalothrin (0.25%) | 43 | 88 | 15-181 | 28-415 |
| Point E | Permethrin (0.4%) | 60 | 151 | 12-815 | 26-2755 |
| Point E | Permethrin (3.75%) | 26 | 45 | 5.45-492 | 8.27-1081 |
| Grand Yoff | Alphacypermethrin (0.08%) | 80 | 237 | 19-706 | 47-2763 |
| Grand Yoff | Alphacypermethrin (0.25%) | 27 | 113 | 5.14-682 | 15-5464 |
| Grand Yoff | Deltamethrin (0.03%) | 98 | 234 | 15-2870 | 29-9663 |
| Grand Yoff | Deltamethrin (0.25%) | 16 | 41 | 7.36-42 | 17-132 |
| Grand Yoff | Lambdacyhalothrin (0.08%) | 68 | 185 | 19-449 | 44-1525 |
| Grand Yoff | Lambdacyhalothrin (0.25%) | 25 | 57 | 12-64 | 25-162 |
| Grand Yoff | Permethrin (0.4%) | 146 | 328 | 4.85-94941481 | 8.07-702694046 |
| Grand Yoff | Permethrin (3.75%) | 21 | 47 | 8.37-77 | 17-204 |
