# Supplementary material for: Entomological surveys and insecticide resistance in the dengue vector Aedes aegypti in Dakar, Senegal: First detection of the kdr mutation
Source: PLoS Negl Trop Dis. 2025 Oct 22;19(10):e0013657. doi: 10.1371/journal.pntd.0013657 (PMC12561948; doi:10.1371/journal.pntd.0013657)
Supplement: S4 Table — (DOCX) [file pntd.0013657.s004.docx]

**S4 Table.** Number of genotypes and frequencies of kdr mutations in the VGSC gene of *Aedes aegypti* from six localities in Dakar

| **Insecticide** | **Study site** | **Phenotype** | **Kdr F1534C** | | | **Allele Freq** |  | **Kdr V1016I** | | | **Allele Freq** | **Kdr V1016G** | | | **Allele Freq** |  | **Kdr S989P** | | | **Allele Freq** |  |  | |
| --- | --- | --- | --- | --- | --- | --- | --- | --- | --- | --- | --- | --- | --- | --- | --- | --- | --- | --- | --- | --- | --- | --- | --- |
|  |  |  |  |  |  |  |  |  |  |  |  |  |  |  |  |  |  |  |  |  |  |  |  |
|  |  |  | CC | FC | FF | F(C) |  | II | VI | VV | F(I) | GG | VG | VV | F(G) |  | PP | SP | SS | F(P) |  | |  |
| perme | Médina | Alive | 0 | 10 | 15 | 0.20 |  | 0 | 0 | 25 | - | 0 | 19 | 6 | 0.38 |  | 1 | 18 | 6 | 0.40 |  | |  |
|  |  | Dead | 0 | 19 | 6 | 0.38 |  | 0 | 0 | 25 | - | 0 | 24 | 1 | 0.48 |  | 3 | 13 | 9 | 0.38 |  | |  |
| Lambda |  | Alive | 0 | 18 | 2 | 0.45 |  | 0 | 0 | 20 | - | 0 | 20 | 0 | 0.50 |  | 0 | 20 | 0 | 0.50 |  | |  |
|  |  | Dead | 0 | 20 | 4 | 0.41 |  | 0 | 0 | 24 | - | 0 | 24 | 0 | 0.50 |  | 1 | 23 | 0 | 0.52 |  | |  |
| Alpha |  | Alive | 0 | 4 | 21 | 0.08 |  | 0 | 0 | 25 | - | 0 | 19 | 6 | 0.38 |  | 1 | 22 | 2 | 0.48 |  | |  |
|  |  | Dead | 0 | 2 | 23 | 0.04 |  | 0 | 0 | 25 | - | 0 | 18 | 7 | 0.36 |  | 0 | 25 | 0 | 0.50 |  | |  |
| Delta |  | Alive | 0 | 8 | 16 | 0.16 |  | 0 | 0 | 24 | - | 0 | 23 | 1 | 0.47 |  | 1 | 23 | 0 | 0.52 |  | |  |
|  |  | Dead | 0 | 3 | 23 | 0.05 |  | 0 | 0 | 26 | - | 0 | 23 | 3 | 0.44 |  | 3 | 23 | 0 | 0.55 |  | |  |
|  |  | Total | 0 | 84 | 110 | 0.21 |  | 0 | 0 | 194 | - | 0 | 170 | 24 | 0.43 |  | 10 | 167 | 17 | 0.48 |  | |  |
|  |  |  |  |  |  |  |  |  |  |  |  |  |  |  |  |  |  |  |  |  |  | |  |
| perme | PE | Alive | 1 | 14 | 9 | 0.33 |  | 0 | 0 | 25 | - | 0 | 23 | 2 | 0.46 |  | 0 | 11 | 13 | 0.22 |  | |  |
|  |  | Dead | 0 | 8 | 17 | 0.16 |  | 0 | 0 | 25 | - | 0 | 25 | 0 | 0.50 |  | 0 | 15 | 10 | 0.30 |  | |  |
| Lambda |  | Alive | 0 | 23 | 2 | 0.46 |  | 0 | 0 | 25 | - | 0 | 24 | 1 | 0.48 |  | 1 | 11 | 13 | 0.26 |  | |  |
|  |  | Dead | 0 | 22 | 3 | 0.44 |  | 0 | 0 | 24 | - | 0 | 23 | 1 | 0.47 |  | 0 | 18 | 7 | 0.36 |  | |  |
| Alpha |  | Alive | 0 | 4 | 20 | 0.08 |  | 0 | 0 | 24 | - | 0 | 24 | 0 | 0.50 |  | 0 | 14 | 10 | 0.29 |  | |  |
|  |  | Dead | 0 | 2 | 24 | 0.03 |  | 0 | 0 | 26 | - | 0 | 26 | 0 | 0.50 |  | 0 | 16 | 10 | 0.30 |  | |  |
| Delta |  | Alive | 1 | 11 | 13 | 0.26 |  | 0 | 0 | 25 | - | 0 | 24 | 1 | 0.48 |  | 0 | 15 | 10 | 0.30 |  | |  |
|  |  | Dead | 0 | 2 | 4 | 0.28 |  | 0 | 0 | 6 | - | 0 | 5 | 1 | 0.41 |  | 0 | 1 | 5 | 0.08 |  | |  |
|  |  | Total | 2 | 86 | 92 | 0.25 |  | 0 | 0 | 180 | - | 0 | 174 | 6 | 0.48 |  | 1 | 101 | 78 | 0.28 |  | |  |
|  |  |  |  |  |  |  |  |  |  |  | - |  |  |  |  |  |  |  |  |  |  | |  |
| perme | Mbao | Alive | 0 | 16 | 9 | 0.32 |  | 0 | 0 | 25 | - | 0 | 25 | 0 | 0.50 |  | 0 | 25 | 0 | - |  | |  |
|  |  | Dead | 0 | 8 | 5 | 0.30 |  | 0 | 0 | 13 | - | 0 | 13 | 0 | 0.50 |  | 0 | 13 | 0 | - |  | |  |
| Lambda |  | Alive | 0 | 4 | 0 | 0.50 |  | 0 | 0 | 4 | - | 0 | 4 | 0 | 0.50 |  | 0 | 4 | 0 | - |  | |  |
|  |  | Dead | 2 | 23 | 0 | 0.54 |  | 0 | 0 | 25 | - | 0 | 25 | 0 | 0.50 |  | 0 | 25 | 0 | - |  | |  |
| Alpha |  | Alive | 0 | 18 | 7 | 0.36 |  | 0 | 0 | 25 | - | 0 | 25 | 0 | 0.50 |  | 0 | 25 | 0 | - |  | |  |
|  |  | Dead | 0 | 10 | 7 | 0.29 |  | 0 | 0 | 17 | - | 0 | 17 | 0 | 0.50 |  | 0 | 17 | 0 | - |  | |  |
| Delta |  | Alive | 0 | 22 | 3 | 0.44 |  | 0 | 0 | 25 | - | 0 | 25 | 0 | 0.50 |  | 0 | 25 | 0 | - |  | |  |
|  |  | Dead | 0 | 16 | 0 | 0.50 |  | 0 | 0 | 16 | - | 0 | 16 | 0 | 0.50 |  | 0 | 16 | 0 | - |  | |  |
|  |  | Total | 2 | 117 | 31 | 0.40 |  | 0 | 0 | 150 | - | 0 | 150 | 0 | 0.50 |  | 0 | 150 | 0 | - |  | |  |
|  |  |  |  |  |  |  |  |  |  |  | - |  |  |  |  |  |  |  |  |  |  | |  |
| perme | Ouakam | Alive | 0 | 1 | 24 | 0.02 |  | 0 | 0 | 25 | - | 0 | 25 | 0 | 0.50 |  | 0 | 25 | 0 | - |  | |  |
|  |  | Dead | 0 | 4 | 20 | 0.08 |  | 0 | 0 | 24 | - | 0 | 24 | 0 | 0.50 |  | 0 | 24 | 0 | - |  | |  |
| Lambda |  | Alive | 0 | 0 | 5 | - |  | 0 | 0 | 5 | - | 0 | 5 | 0 | 0.50 |  | 0 | 5 | 0 | - |  | |  |
|  |  | Dead | 0 | 0 | 25 | - |  | 0 | 0 | 25 | - | 0 | 25 | 0 | 0.50 |  | 0 | 25 | 0 | - |  | |  |
| Alpha |  | Alive | 1 | 3 | 21 | 0.10 |  | 0 | 0 | 25 | - | 0 | 25 | 0 | 0.50 |  | 0 | 25 | 0 | - |  | |  |
|  |  | Dead | 0 | 2 | 23 | 0.04 |  | 0 | 0 | 25 | - | 0 | 25 | 0 | 0.50 |  | 0 | 25 | 0 | - |  | |  |
| Delta |  | Alive | 0 | 3 | 19 | 0.06 |  | 0 | 0 | 22 | - | 0 | 22 | 0 | 0.50 |  | 0 | 22 | 0 | - |  | |  |
|  |  | Dead | 0 | 0 | 16 | - |  | 0 | 0 | 16 | - | 0 | 16 | 0 | 0.50 |  | 0 | 16 | 0 | - |  | |  |
|  |  | Total | 1 | 13 | 153 | 0.04 |  | 0 | 0 | 167 | - | 0 | 167 | 0 | 0.50 |  | 0 | 167 | 0 | - |  | |  |
|  |  |  |  |  |  |  |  |  |  |  |  |  |  |  |  |  |  |  |  |  |  | |  |
| perme | Grand Yoff | Alive | 0 | 6 | 19 | 0.12 |  | 0 | 0 | 25 | - | 0 | 25 | 0 | 0.50 |  | 0 | 25 | 0 | - |  | |  |
|  |  | Dead | 0 | 1 | 18 | 0.02 |  | 0 | 0 | 19 | - | 0 | 19 | 0 | 0.50 |  | 0 | 19 | 0 | - |  | |  |
| Lambda |  | Alive | 0 | 15 | 10 | 0.30 |  | 0 | 0 | 25 | - | 0 | 25 | 0 | 0.50 |  | 0 | 25 | 0 | - |  | |  |
|  |  | Dead | 1 | 7 | 17 | 0.18 |  | 0 | 0 | 25 | - | 0 | 25 | 0 | 0.50 |  | 0 | 25 | 0 | - |  | |  |
| Alpha |  | Alive | 0 | 13 | 11 | 0.27 |  | 0 | 0 | 24 | - | 0 | 24 | 0 | 0.50 |  | 0 | 24 | 0 | - |  | |  |
|  |  | Dead | 0 | 9 | 14 | 0.19 |  | 0 | 0 | 23 | - | 0 | 23 | 0 | 0.50 |  | 0 | 23 | 0 | - |  | |  |
| Delta |  | Alive | 0 | 2 | 23 | 0.04 |  | 0 | 0 | 25 | - | 0 | 25 | 0 | 0.50 |  | 0 | 25 | 0 | - |  | |  |
|  |  | Dead | 0 | 0 | 6 | - |  | 0 | 0 | 6 | - | 0 | 6 | 0 | 0.50 |  | 0 | 6 | 0 | - |  | |  |
|  |  | Total | 1 | 53 | 118 | 0.15 |  | 0 | 0 | 172 | - | 0 | 172 | 0 | 0.50 |  | 0 | 172 | 0 | - |  | |  |
|  |  |  |  |  |  |  |  |  |  |  |  |  |  |  |  |  |  |  |  |  |  | |  |
| perme | Guédiawaye | Alive | 0 | 0 | 25 | - |  | 0 | 0 | 25 | - | 0 | 19 | 6 | 0.38 |  | 0 | 25 | 0 | - |  | |  |
|  |  | Dead | 0 | 0 | 14 | - |  | 0 | 0 | 14 | - | 0 | 0 | 14 | -- |  | 0 | 14 | 0 | - |  | |  |
| Lambda |  | Alive | 0 | 0 | 15 | - |  | 0 | 0 | 15 | - | 0 | 15 | 0 | 0.50 |  | 1 | 14 | 0 | 0.53 |  | |  |
|  |  | Dead | 0 | 0 | 25 | - |  | 0 | 0 | 25 | - | 0 | 25 | 0 | 0.50 |  | 1 | 0 | 24 | 0.04 |  | |  |
| Alpha |  | Alive | 0 | 0 | 21 | - |  | 0 | 0 | 21 | - | 0 | 21 | 0 | 0.50 |  | 0 | 21 | 0 | - |  | |  |
|  |  | Dead | 0 | 0 | 16 | - |  | 0 | 0 | 16 | - | 0 | 15 | 1 | 0.46 |  | 1 | 15 | 0 | 0.53 |  | |  |
| Delta |  | Alive | 0 | 0 | 24 | - |  | 0 | 0 | 24 | - | 0 | 24 | 0 | 0.50 |  | 0 | 24 | 0 | - |  | |  |
|  |  | Dead | 0 | 0 | 7 | - |  | 0 | 0 | 7 | - | 0 | 7 | 0 | 0.50 |  | 0 | 7 | 0 | - |  | |  |
|  |  | Total | 0 | 0 | 147 | - |  | 0 | 0 | 147 | - | 0 | 126 | 21 | 0.42 |  | 3 | 120 | 24 | 0.42 |  | |  |
