# Supplementary material for: Entomological surveys and insecticide resistance in the dengue vector Aedes aegypti in Dakar, Senegal: First detection of the kdr mutation
Source: PLoS Negl Trop Dis. 2025 Oct 22;19(10):e0013657. doi: 10.1371/journal.pntd.0013657 (PMC12561948; doi:10.1371/journal.pntd.0013657)
Supplement: Attachment — Submitted filename: Reply to the editor and reviewers Comments.docx [file pntd.0013657.s006.docx]

                                                                                             Dakar, 06th October 2025

Dear Editor of PLOS Neglected Tropical Diseases

Ref: Research paper revised (Manuscript PNTD-D-25-00521R1)

We thank the editor and all the reviewers for their comments and suggestions on the current manuscript titled **“Entomological Surveys and Insecticide Resistance in the Dengue Vector *Aedes aegypti* in Dakar, Senegal: First Detection of the *kdr* Mutation”**. In the minor revision manuscript, we have addressed all the comments and recommendations. All changes are highlighted in the main document. Kindly find the attached below point-by-point responses to the comments.

Yours Sincerely,

Corresponding authors:

Dr. Ndeye Marie Sene;  ndeyemarie.sene@pasteur.sn ; marie2019@hotmail.fr

**Point by point responses to Reviewer(s)' Comments to Author:**

This is a significantly improved version of the MS. Please take note of the following:
Line 31. Kdr mutations should be fully written for the first time.

**Response: Revised as suggested on See L31**

**“*Molecular screening targeted* knockdown resistance (kdr) mutations *(F1534C, V1016G/I, S989P).”***

Line 33: Please use the lowercase "dengue."

**Response: Revised as suggested on See L34**

**“dengue virus (DENV)”**

Line 41: It would be helpful to briefly list the specific multiple resistance mechanisms that were detected here.

**Response: listed as suggested. See L42-45**

**“In this study, we detected three kdr mutations (F1534C, V1016G, and S989P), while previous studies in Senegal have documented metabolic resistance mechanisms. Together, these resistance mechanisms may compromise the efficacy of vector control strategies”**

Lines 121-124. The coexistence of kdr mutations and metabolic resistance mechanisms has also been reported in sub-Saharan Africa (Montgomery et al., 2022)

**Response: Revised as suggested on L121-123**

**“The coexistence of *kdr* mutations and metabolic resistance mechanisms, has been widely documented in *Ae. aegypti* populations across** **Southeast Asia, sub-Saharan Africa and Latin America [17–20].”**

Lines 130-131. The objective of this specific part of the study could be stated more explicitly for the reader.

**Response: Revised as suggested. See L130-134**

**“The objectives of this study were to: (i) establish baseline data on the distribution and behavior of *Ae. aegypti* populations, including egg density over space and time, host-seeking, host-preferences, and resting behavior; (ii) assess the infection rates of collected mosquitoes with arboviruses; and (iii) characterize the insecticide resistance status and resistance mechanisms in the populations across various study sites.”**

Line 194. CO2 include a subscript

**Response: We corrected the typography on L195**

**“CO₂”**

Line 216-217. “The mosquitoes were homogenized and used for RNA extraction and Blood Feeding Patterns”. This sentence is not clear.

**Response:	We reworded to clarify. See L217-221**

**« Each pool of *Ae. aegypti* mosquitoes (whole mosquitoes for non-engorged and heads and thoraxes for engorged females) was homogenized in L-15 medium (GibcoBRL, Grand Island, NY, USA) for RNA extraction and arbovirus detection. For engorged females, the abdomens were homogenized in Phosphate-Buffered Saline (PBS), and the homogenate was used to determine the origin of their blood meals.”**

Please clarify the quality/standard of the insecticide-impregnated papers used, including the justification of concentration values used.

**Response: We reworded to clarify. See L287-299**

**“The Vector Control Research Unit, Universiti Sains Malaysia (WHO collaborating Centre) provided all insecticide-impregnated papers used in this study. Tested insecticides papers included: pyrethroids (0.4% and 3.75% permethrin, 0.03% and 0.25% deltamethrin, 0.08% and 0.25% lambda-cyhalothrin, 0.08% and 0.25% alpha-cypermethrin), carbamates (0.2% bendiocarb) and organochlorines (1.5% malathion, 60 mg/m2 pirimiphos-methyl and 1.0% chlorpyriphos-ethyl). The lower concentrations used correspond to the WHO-recommended new diagnostic doses for assessing *Aedes* susceptibility. The higher concentrations (pyrethroids 3.75% permethrin, 0.25% deltamethrin 0.25% lambda-cyhalothrin and 0.25% alpha-cypermethrin) are not currently the standard for *Aedes* but are usually applied in *Anopheles* intensity essay. They were used here due to the availability of pre-impregnated papers and to provide initial data one the populations under study. Papers impregnated with olive oil (for organophosphate and carbamate control), and silicone oil (for pyrethroid control) were used as control. For pyrethroids, cumulative knockdown rates were recorded after 10, 20, 30, 40, 50, 60, 70 and 80 min. After 1-hour exposure period, mosquitoes were transferred to holding tubes, provided with 10% sugar solution, and kept at 25°C (± 2°C) and 70% (±10%) relative humidity. Mortality was recorded at 24-hours post-exposure. ”**

The legend of Figure 3 is incomplete.

**Response: The legend was completed as suggested. See L399-401**

**“Fig 3. Mean number of host-seeking Aedes aegypti females collected using Biogents (BG) Sentinel traps in Dakar from August 2022 to July 2023. Error bars indicates 95% CI: 95% Confidence interval.”**

Table 4. The legend is incomplete. CC, PP, II, CF, FF, VI, Freq ….

**Response: Completed as suggested. See L510-512**

**CC, II, VV, and SS = homozygous wild-type; FC, VI, VG, and SP = heterozygous (mutant/wild-type); FF, II, GG, and PP = homozygous mutant. Freq = frequency of the mutant allele.**

Line 592-592. Could you specify which of the insecticides listed in line 591 are commonly used for household nuisance control in Dakar? A reference for this common usage would be ideal here.

**Response: Revised as suggested. See L603-605**

**“In particular, pyrethroids such as permethrin, deltamethrin, and allethrin are widely used in Dakar households for nuisance mosquito control in the form of sprays, coils, and aerosols [6]. Carbamates such as propoxur are used in reactive intervention campaigns during epidemics, and resistance to this class of insecticides (bendiocarb) has been observed [7].”**

For context, please include the approximate distance between your study sites in Dakar and the Niayes zone. Also, a brief note on the known dispersion capacity of Aedes mosquitoes would strengthen the discussion.

**Response: Revised as suggested. See L607-612**

**“This area is, characterized by intensive urban agriculture, particularly market gardening, which involves frequent use of organophosphates and pyrethroids [51]. Ae. aegypti is characterized by a limited flight range, generally not exceeding 100–200 meters under natural conditions [52], although some individuals have been reported to travel up to 800 meters [53].”**

Lines 638-641. It would be good to add the reference of studies reporting 410 mutation in Africa

**Response: Revised as suggested. See L657**

**“One limitation of this study is that the V410L kdr mutation, which has been increasingly reported in Ae. aegypti populations across West Africa, was not included in our screening panel [11,14,15].”**
